# Alcohol Consumption Reported during the COVID-19 Pandemic: The Initial Stage

**DOI:** 10.3390/ijerph17134677

**Published:** 2020-06-29

**Authors:** Jan Chodkiewicz, Monika Talarowska, Joanna Miniszewska, Natalia Nawrocka, Przemyslaw Bilinski

**Affiliations:** 1 Institute of Psychology, University of Lodz, 91-433 Lodz, Poland; monika.talarowska@uni.lodz.pl (M.T.); joanna.miniszewska@uni.lodz.pl (J.M.); natalia.nawrocka91@gmail.com (N.N.); 2 The President Stanisław Wojciechowski State University of Applied Sciences in Kalisz, 62-800 Kalisz, Poland; bildom@gmail.com; 3 Copernicus Memorial Multidisciplinary Comprehensive Cancer and Traumatology Center, 91-433 Lodz, Poland

**Keywords:** alcohol, mental health, stress, COVID-19

## Abstract

Physical health is not the only area affected by the outbreak of the SARS-CoV-2 virus pandemic. There are also other consequences that have globally affected many millions at other levels, namely: Societal, political, economic, and cultural. This study aims to survey alcohol drinking throughout the pandemic so as to investigate those factors considered most relevant; i.e., sociodemographic and clinical. A longitudinal study was designed. The first (or initial) stage was completed between April 10–20 2020 on 443 subjects during the enforcement of the “Lockdown” in Poland. The second stage will be due in June 2020. As well as an in-house questionnaire, the study used: The Alcohol Use Disorder Identification Test (AUDIT), General Health Questionnaire (GHQ-28), Perceived Stress Scale (PSS-10), and the Brief COPE Inventory (Mini COPE). Alcohol was the most commonly used psychoactive substance (73%) identified. More than 30% changed their drinking habits because of the pandemic, with 16% actually drinking less, whilst 14% did so more. The former group was significantly younger than the latter. Amongst the stress-related coping strategies, it was found that current alcohol drinkers were significantly less able to find anything positive about the pandemic situation (positive reframing) and were mentally less able to cope. Those drinking more now were found to have been drinking more intensively before the pandemic started.

## 1. Introduction

Billions of people had their lives dramatically changed when the SARS-CoV-2 virus epidemic emerged in 2020, (termed “severe acute respiratory syndrome coronavirus”). The outbreak started in December 2019 in the city of Wuhan in Central China. By 11 March 2020, the World Health Organization (WHO) had declared it a pandemic [1]. The coronavirus illness (COVID-19) is a highly contagious and infectious disease, most often causing fever, fatigue, dry cough, muscle pain, and shortness of breath. What distinguishes this current pandemic from others is its very fast spread and relatively high mortality. This was confirmed in mid-March 2020 when cases were recorded in 135 countries in all continents, roughly 3 months after the first cases had emerged. On March 1, mortality rates reached 3.6% in China and 1.5% outside of China [2].

Figures provided by the WHO on 8 May2020 attest to the rapid spread of the virus, with over 3.8 million infections confirmed worldwide and 270,000 deaths [3]. Unprecedented measures were adopted to contain the spread of virus, which caught the healthcare systems in many countries unawares and disorganized; including highly developed countries such as Italy, the USA, and Spain. Because there are no pharmacological agents currently available for effective treatment, interventions have focused on limiting social contact, the quarantine of persons suspected of having contact with the virus, enforcing social distancing (closing shops, cinemas, theaters, restaurants) and conducting media campaigns regarding the best means for protecting against infection (i.e., masks, hand washing, decontamination) [4].

We do not however know exactly how long the pandemic will last, nor its dynamics of development despite all the countermeasures introduced in February and March 2020, and the subsequent relaxation of some of these later. The economic impact of the restrictions imposed also remains unknown [5].

The pandemic situation is undoubtedly a crisis felt by huge numbers of people, leading to difficult psychological consequences. It takes considerable effort to re-adapt to an unknown and uncertain situation and how to deal with many unpleasant emotions, daily irritations, and the prospect of a threatened material existence to oneself and family.

Due to the well-known and documented effects of inhibiting the nervous system, alcohol and other psychoactive substances are used by many people seeking relief from unpleasant emotions, stress, anxiety, or depression [6,7,8]. This begs the question of whether the significant rises in alcohol sales, (including those by mail), seen in many countries are due to the pandemic when compared to the same period in the previous year. As an example, a March 2020 study conducted by the USA Nielsen Company found 240% increases in internet alcohol sales, including strong liquors (spirits) by 75%, wines by 66%, and beers by 42% [9]. There have also been press reports of increased domestic violence, which has long been associated with alcohol abuse [10]. Nevertheless, alcohol sales are not in themselves reliable enough estimates of alcohol consumption, because in these present circumstances, restaurants, clubs, and pubs have been all shut, i.e., those places where many people have been used to regularly drinking alcohol; especially young people.

Reports from the scientific literature are however equivocal on this issue. There have been increased trends shown in alcohol consumption, drunkenness, and alcoholic excesses during economic crises (e.g., the 2007–9 recession) [11,12,13]. Further reports confirm increased alcohol consumption following other dramatic events, such as Hurricane Katrina, Hurricane Rita, and the attack on the World Trade Center [14]. In contrast, it ought to be stressed that economic crises reduce alcohol consumption in some people, mainly due to financial problems or the risk of losing one’s job because of continuing to excessively drink [11,15].

Nonetheless, the current crisis differs from the ones aforementioned. This is not only an economic issue or a dramatic one-off event, but a complex multi-faceted experience affecting billions of people worldwide in spheres such as: Medical, social/societal, political, geopolitical, economic, religious, cultural, axiological, and civilizational dimensions. Therefore, this covers almost all areas in the lives of individuals and societies, including [16]. It is thereby of great interest to understand how/why psychoactive substances, especially alcohol, are used in this challenging time. Our study has aimed to answer such questions on alcohol consumption during the pandemic and to investigate other associated factors like the sociodemographic and clinical ones. Of particular interest were the relationships between alcohol consumption with mental stress and coping strategies.

## 2. Materials and Methods

The study was designed to be two-staged and longitudinal. The first part was carried out in April 10–22, 2020, when the lockdown had been in effect for one month, whereas the second stage is pending from June 2020. Because of the epidemiological conditions of the pandemic, the study was performed online on adult subjects recruited by the “Snowball” method and conducted using Google Form, obtained inter alia via Facebook. The full procedures and required links can be found in publications from PARPA, the Polish Psychiatric Association, and others.

Subjects for the first stage consisted of n = 443 adults of whom 348 were women (78.6%) and 95 men (21.4%), with a mean overall age of 31.9 years (SD = 11.31 and range of 18–68 years). The sociodemographic breakdown is shown in Table 1.

Table 1 shows that most subjects were large-city dwellers, possessed higher education, no offspring, and worked full-time. The numbers of single to married persons were almost evenly matched.

### Study Tools

Alcohol use disorder was diagnosed by the Alcohol Use Disorder Identification Test (AUDIT) developed by the World Health Organization [17]. This test was developed as a screening tool, to identify people whose alcohol consumption has become risky (hazardous), harmful, or addictive. The method is based on multi-country studies which indicate that certain symptoms (e.g., high alcohol consumption, alcohol-related violation of social norms, alcohol-related injuries) may constitute early warning signs of an alcohol problem and a developing addiction. As an attempt to identify those symptoms that would be common to different countries and cultures, the test consisted of three questions on the amount and frequency of drinking (Nos 1–3), three about alcohol addiction (Nos 4–6), and four on the problems caused by alcohol (Nos 7–10). Scoring grades from these 10 questions were as follows: 8–15 points (pts) indicates likely hazardous drinking, 16–19 pts harmful drinking, and 20 pts or more a likely alcohol addiction. This has been checked across many countries and cultures by comparing the AUDIT results together with external measures, including the opinions of specialists [17].

When choosing other study tools, we focused on those that are designed to provide a recent analysis of a respondent’s wellbeing, i.e., within a previous month or less. For this reason, we used the following:

1. A Polish-adapted Goldberg General Health Questionnaire (GHQ-28) was used to measure mental health [18], which consisted of 28 questions divided into four scales of seven questions each. They concerned the subject’s well-being over the past few weeks. Four aspects of mental health were appraised: (1) Somatic symptoms, (2) anxiety and insomnia, (3) dysfunction in everyday life, and (4) depression symptoms. Each scale scoring ranged between 0–21 points and their sums gave a general measure on the state of mental health. The reliability of the method using Cronbach-α in the present study ranged from 0.79 (somatic symptoms) to 0.91 (depression scale). The reliability of the overall measure was 0.94.

2. Stress levels were measured by the Polish version of Cohen’s, Kamarck’s, and Mermelstein’s Perceived Stress Scale (PSS 10) [19]. The questionnaire comprised 10 questions for assessing the intensity of stress related to one’s own life situation during the last month. Respondents used a 5-point frequency scale (0–4) for giving their answers ranging from the “never” to “very often” categories. The method was satisfactorily reliable (Cronbach’s α 0.84) and any Polish sten scores that were 7 or above and any results above 20 pts were considered to be high.

3. Coping strategies were measured by the Polish version of the Carver MINI-COPE Questionnaire (Brief COPE Inventory) [20]. There are 28 statements integrated into 14 coping strategies (i.e., two statements per strategy), these being as follows: Active coping, planning, positive reframing, acceptance, humor, religious solace, use of emotional support, use of instrumental support, self-distraction, denial, venting, substance abuse, behavioral disengagement, and self-blame. The respondent selected one out of four possible replies ranging in scores from “I have almost never been doing this” (0 points) to “I have almost always been doing this” (3 points). Each of the coping strategies was assessed separately and the higher the score, then the more often a particular strategy was adopted.

The method can be used to assess coping by either flexible or situational approaches. Our study used the second option (i.e., coping with a pandemic situation). Internal compliance of the questionnaire was 0.86. Another questionnaire was also given regarding the use of psychoactive substances on one’s mental and somatic health, and on behavior during the pandemic.

Statistical analyses consisted of the Student’s *t*-test, analysis of variance (ANOVA), and a chi square test performed on the STATISTICA program software.

## 3. Results

Replies to questions on psychoactive substance abuse were firstly analyzed, and a breakdown is presented in Table 2.

As expected, alcohol proved to be most commonly used of the psychoactive substances (almost 73%), followed by smoking tobacco (just less than 25%) and then by recreational drugs (almost 4%). Over 30% of respondents had also changed their drinking habits during the pandemic, with some drinking less, some more; respectively 16% and 14%.

The AUDIT test outcomes gave an M value of 5.80 (SD = 4.05) which reflect the average level of alcohol use. A more detailed comparison of the results with specified test standards showed that 125 subjects (28.22%) were graded into hazardous drinking, 3 subjects into harmful drinking (0.7%), and 4 into a possible addiction (0.9%). The remaining 311 (70.2%) were either abstinent (27%) or drank at low risk levels. There were no significant differences found between the hazardous drinking and low risk drinking groups according to the tested variables of stress levels, mental health levels, and almost all of the coping strategies. The only difference was in the denial coping strategy, where hazardous drinking subjects used this strategy more often (M = 1.11, SD = 1.43 versus M = 0.73, SD = 1.11; t = 3.39, p < 0.01; and Cohen d of 0.35; moderate effect).

In keeping with the study aims, the following comparisons between subject groupings before and during the pandemic were thus made according to sociodemographic and clinical variables: Those with the same drinking behavior (Group 1, n = 182), those drinking less (Group 2, n = 77), those drinking more (Group 3, n = 61), and abstainers (Group 4, n = 123). This also included the data obtained from using the study tools. 

There was no significant effect of gender found in any of the relationships between the sociodemographic variables, nor in the grades of alcohol behavior during the pandemic; p = 0.15. Of those in permanent relationships (n = 203), 34 declared they drank more alcohol (7.74%), whilst in the singles group (n = 209) only 21 subjects did so (4.78%). Subjects in permanent relationships who drank less were n = 19 (4.33%), those having unchanged drinking habits before and during the pandemic were n = 94 (21.41%), whilst those who currently drank less were n = 56 (12.76). Such differences were statistically significant (p < 0.001), meaning that there were more subjects drinking intensively when being in a relationship than when single, compared to before the pandemic started.

Out of those subjects (n = 301) with offspring, 39 (8.88%) declared currently drinking more than before the pandemic, whilst 69 (15.72%) in this respect drank less. In those without children (n = 138) the opposite holds true, where 5% drank more now than before the pandemic; this being statistically significant at p < 0.001.

Most subjects had received higher and upper level secondary education (n = 427) and it was found that education levels made no significant impact on their grouping differentiation; p = 0.12. Of those with higher education (n = 259), 38 (8.66% of all subjects) drank more alcohol recently, as likewise did subjects with upper level secondary education; n = 21 (4.78% of all subjects). Because there were so few subjects educated at lower levels, (n = 12 in all; 2–7%) it was not possible to make any meaningful estimates.

Most subjects lived in cites of >100K inhabitants (n = 273). There were, however, no significant effects of where people lived, (i.e., city/town population sizes), on any of the outcome rates; p = 0.818.

We found that being variously engaged in work during the pandemic did not make any difference to alcohol consumption rates; p = 0.277; however, admittedly, the unemployed group was rather small at n = 24. There were also no significant differences found in drinking alcohol between how people otherwise spent their day, whether by sporadically going out (e.g., shopping, walking the dog), staying at home, or going out to regular work all the time (n = 88); p = 0.262. Even those in quarantine (n = 38; 8.6%) did not significantly increase their alcohol intake from the others; p = 0.104. 

The clinical results demonstrate that 134 subjects (30.52%) had been treated for mental disorders before the pandemic began, of whom 49 subjects were currently on pharmacotherapy, whilst 41 had always been so previously. Just by being on such treatment had no effect on altering alcohol consumption rates; p = 0.284. We found that 47 subjects (10.71%) who had currently entertained suicidal thoughts and who were now much more likely to drink more alcohol than those without such thoughts before the pandemic started; p = 0.024. Somatic illnesses were noted in 50 subjects (11.3% of total), and their alcohol drinking rates were less than those who were healthy; p = 0.006. As many as 222 respondents (50.57%) declared that alcohol addiction was present in their families. Interestingly, this group of subjects currently consumed significantly less alcohol than in those respondents from families devoid of alcohol problems; p = 0.04.

Table 3 presents differences in patterns of drinking found in subjects according to age, and outcomes from the psychometric tools used during the pandemic. 

Table 3 shows a row of variables differentiated into groups of alcohol consumption. Subjects declaring low alcohol consumption were significantly younger (at a mean of about 26 years) than the rest (mean above 30 years). Those admitting to having a higher current alcohol consumption achieved higher mean results on the AUDIT scale, signifying that their alcohol consumption was already intensive and frequent prior to the pandemic, when compared to those currently drinking either at levels unchanged before the pandemic or who were abstaining. 

It was seen that subjects who drank more alcohol were significantly less likely to derive any positive benefits from their stress coping strategies during the pandemic situation (positive reframing). In the light of the analyzed variables, the differences observed in the use of psychoactive substances are hardly surprising. People who currently drank more scored significantly higher when adopting this strategy (p < 0.001) than those who drank the same before the pandemic started and likewise to those drinking less. This may signify that they have simply adopted drinking alcohol as a strategy for coping with difficult situations in general, not just the current one.

Subjects who were now drinking more than before the pandemic started also possessed worse mental health than the other groups; i.e., they coped less well with everyday functioning and in doing their daily tasks and duties; deriving less satisfaction with their actual performance (functional disorders). They also significantly suffered more from depression, i.e., the sense that life is hopeless, a low self-esteem, and suicidal thoughts.

Finally, the ways that alcohol was abused was compared in those scoring highly in the GHQ 28 questionnaire (i.e., 9–10 sten scores according to Polish standards), thereby strongly indicating the prospect of serious mental disorders arising (n = 16, 26.2%). Furthermore, significantly more respondents in this group were drinking alcohol more intensely than before their therapy had started, compared to those with the lower scores; p = 0.003.

## 4. Discussion of Results

The pandemic situation now confronting humankind can be considered to be rather a complex and multiple-stage crisis, affecting the many aspects of health, including mental health; in both societal and individual dimensions [21]. Notwithstanding the threat posed by the virus itself, various psychological problems may arise from the enforced lockdown and isolation (i.e., quarantine) along with economic threats. Animal studies have inter alia demonstrated the negative impact that isolation has on increasing body stress levels with both elevated neuroendocrine responses and reactivity to stress [22]. All this can elicit a wide spectrum of disorders of varying severity, particularly problems in concentrating, anxiety, depression, insomnia, aggression, and interpersonal conflicts. The intensity of these symptoms may at least in part be due to the duration and extent of the quarantine, a feeling of loneliness, fear of infection, and access to appropriate or inappropriate information [23].

For these reasons, one of the challenges currently facing investigators is in monitoring mental health problems, which can help in designing and executing the appropriate remedial interventions [24]. 

The present study purposed to determine the use of psychoactive substances, especially alcohol, during the pandemic and to elucidate the factors associated with this phenomenon; taking into account a broad spectrum of variables. Like Clay and Parker [22], it was assumed that the effects of the crisis and isolation would also be felt in the area of alcohol consumption and abuse. We investigated 443 subjects using extensive surveys and research tools of confirmed psychometric value. Such tools were selected so that they were suitable for analyzing recent occurrences; usually within the last month.

When regarding alcohol consumption, the study showed that more than 28% of respondents drank at-risk levels, and almost the same number maintained abstinence. This proportion found of at-risk drinking is higher than in population studies, both Polish and American [25,26,27]. According to the data of the State Agency for the Prevention of Alcohol-Related Problems (PARPA), the number of harmful drinkers is about 2–2.5 million (5–7% of the population), and risky drinkers over 11% [25]. At this stage, it is difficult to say whether the stress situation associated with the pandemic or the specificity of the studied group is responsible for the noted differences. At this stage, it is also difficult to say whether this is due to the stressful situation associated with the pandemic or the specificity of the studied group.

To meet the study aims, it was important to compare subjects who now drank more alcohol since the start of the pandemic with those who drank less or were drinking as before. The study shows that younger respondents mainly drank less alcohol if they were single and didn’t have any children (these variables were joined together). This may be because pubs, clubs, and halls of residence are now all closed, and classes at schools and colleges are suspended, thus preventing (or lessening the opportunity of) contact and meetings between young adults as they are now staying at home. A lowered alcohol consumption may thus be the result of necessity as opposed to an informed decision. The question remains on whether this consumption will change after the lifting of sanctions.

Those most vulnerable to developing alcohol abuse and other problems are the subjects who reported drinking more alcohol from the time the pandemic started; less than 14% of the total. Our analyses demonstrated the following features in these subjects:

- Intensive alcohol drinking even before the pandemic began (high AUDIT score);
- They rarely or never adopted the positive reframing strategy for coping, but instead more frequently used substances to react to stress;
- Currently having a worse state of mental health, difficulties in coping with everyday activities, depressed mood and other symptoms of depression, as well as harboring thoughts of suicide;
- Actually experiencing suicidal thoughts in a given situation.

Subjects fulfilling these criteria appeared to be at the highest risk of developing alcohol-related and other mental health problems. It is highly likely that they turn to alcohol for “self-healing” their mental problems, however this has been long known to bear a high health risk [7]. This behavior is all the more dangerous, as a number of pre-pandemic studies have shown bilateral associations between depressive symptoms and suicidal tendencies linked with alcohol problems, including [28,29]. Those harboring suicidal thoughts particularly merited attention; not only in the possible problems of alcohol abuse, but that the crises arising to date have actually led to significant increases of suicide as documented in UK, Greek, and USA studies (a 60% rise during the pandemic) [30,31,32]. Young adults also deserve additional attention because of particularly strong relationships observed between poor mental health with depression and coping with these states by drinking alcohol resulting in the development of problem drinking and alcohol-related accidents [33,34].

Subjects drinking more intensively during the pandemic than before likewise often preferred to deal with stressful experiences by using stimulants, but to a lesser extent through positive reframing. It is worth noting that the first way of dealing with stress is widely considered to be non-adaptive, whereas the second is highly adaptive (i.e., strategies also investigated by our study tools), including [35]. Many studies have demonstrated positive relationships between maladaptive stress coping strategies and the development of alcohol-related problems, together with the protective role of adaptive strategies, including [36].

The second part of our study should be able to answer how these subjects function after more time has elapsed.

The presented studies have their limitations. Firstly, there is an over-representation of women and young people that are employed, have higher and upper-level secondary education, and live in large cities. Consequently, our results clearly cannot be used to generalize for the entire population. Furthermore, there were hardly any harmful drinkers and addicts (only 7 subjects), and thus the important question about how such people cope during the present crisis will remain begging, especially in times of high stress when access to therapy and AA (Alcoholics Anonymous) meetings are limited. Separate studies are needed to investigate this issue. Future studies should not only focus on coping strategies, but on the role that supporting social services play for preventing the development of alcohol problems. USA studies have proved that crisis-related job loss was significantly linked to alcohol use disorders; predominantly amongst African-Americans, much less in Caucasians, whilst there were no such links observed with Latinos. One way of explaining this discrepancy was in assuming different levels of social support in these groups, which by these means have been proven to afford protection [37].

## 5. Conclusions

Nearly 30% of subjects consumed at-risk quantities of alcohol—this result is significantly higher than data on the Polish population. In addition, 14% of respondents increased their alcohol consumption since the onset of a pandemic. For all these people, it seems advisable to conduct appropriate information and preventive campaigns, as well as further research on the structure of their alcohol consumption.

In those subjects who increased their alcohol intakes during the pandemic (and so being vulnerable to alcohol-related problems), the majority had drunk alcohol intensively before it started and suffered from worse mental health. They were probably unaware of any positive effects of the current situation and appeared to prefer using substances as a previously tried way of managing and alleviating their stress. Their levels of mental health were also reduced, especially in daily functioning and symptoms of depression.

Sociodemographic variables show weak relationships (or lack of them) with drinking patterns during a pandemic. Gender, education, employment, and place of residence (city, village) do not differentiate the way of drinking. People who drink more are older, somatically healthy, and have no children. This phenomenon requires further research.

## Figures and Tables

**Table 1:** Sociodemographic breakdown of Stage 1 subject group.

	N = 443	%
Place of residence		
Village	69	15.58
Town < 100K inhabitants	100	22.57
City > 100K inhabitants	274	61.85
Education		
Primary/middle school	8	1.81
Vocational	4	0.90
Secondary upper level	170	38.37
Higher	261	58.92
Marital status		
Single	210	47.40
Married/partner	204	46.05
Divorced/separated	27	6.09
Widowed	2	0.45
Children		
Yes	140	31.60
No	303	68.40
Employment		
Studying/training	93	20.99
Studying/working	79	17.83
Working full-time	225	50.79
Casual work	21	4.74
Unemployed	25	5.64

**Table 2:** Psychoactive/medicinal substance use and observed changes during the pandemic.

	N = 443	*%*
Do you drink alcohol?		
Yes	323	72.9
No, I am an abstainer	120	27.1
Do you smoke tobacco, use e-cigarettes or heated tobacco?		
Yes	109	24.6
No	334	75.4
Do you currently take any prescribed sedatives or sleeping pills?		
Yes	38	8.6
No	405	91.4
Do you currently take any non-prescribed (OTC) sedatives or sleeping pills?		
Yes	28	6.3
No	415	93.7
Do you currently use any recreational drugs (e.g., marijuana, amphetamines) or so-called designer drugs (legal highs) (e.g., mephedrone)?		
Yes	16	3.6
No	427	96.4
If you drink alcohol, has your consumption changed since the introduction and enforcement of the Coronavirus lockdown?		
No, it’s the same	182	41.1
Yes, I drink less	77	17.4
Yes, I drink more	61	13.8
No, I am an abstainer	123	27.8
If you smoke tobacco, has your smoking changed since the introduction and enforcement of the Coronavirus lockdown?		
No, it’s the same	65	14.6
Yes, I smoke less	22	4.9
Yes, I smoke more	26	5.8
I am a non-smoker	330	74.4
If you use recreational drugs, has your consumption changed since the introduction and enforcement of the Coronavirus lockdown?		
No, it’s the same	17	3.8
Yes, I take less	5	1.1
Yes, I take more	6	1.4
I don’t take drugs	415	93.7

**Table 3:** A comparison of drinking patterns in subjects during the pandemic.

	Group 1 N = 182		Group 2 N = 77		Group 3 N = 61		Group 4 N = 123		F	Post-Hoc Test
	M	SD	M	SD	M	SD	M	SD		
Age	32.39	10.43	26.77	8.92	31.54	11.45	34.61	12.80	8.02	2 < 1,3,4
AUDIT	4.83	3.627	5.701	3.671	7.049	4.853	5.520	4.272	4.77	3 > 1,4 *
PSS 10	18.45	6.931	18.77	6.782	20.64	6.463	19.03	6.099	1.65	-
MINI COPE active coping	3.28	1.68	3.06	1.51	2.79	1.71	3.41	1.70	2.36	-
Planning	3.91	1.56	3.47	1.52	3.62	1.59	3.90	1.53	1.93	-
Positive reframing	3.75	1.74	3.32	1.69	2.97	1.90	3.77	1.67	4.13	3 < 1,4
Acceptance	4.76	1.24	4.68	1.13	4.44	1.69	4.65	1.29	0.90	-
Humor	2.50	1.31	2.42	1.22	2.54	1.35	2.00	1.28	4.12	1, 3 > 4
Religion	1.42	1.79	1.10	1.56	1.03	1.67	2.14	2.13	7.53	4 > 1,2,3
Use of emotional support	3.96	1.69	3.44	2.06	4.02	1.73	3.56	1.76	2.49	-
Use of instrumental support	3.40	1.68	3.16	1.90	3.44	1.87	3.17	1.71	0.73	-
Self-distraction	3.92	1.43	3.84	1.30	3.48	1.58	3.80	1.36	1.51	-
Denial	0.65	1.08	0.78	1.23	0.80	1.34	1.03	1.35	2.43	4 > 1
Venting	3.23	1.55	3.21	1.73	3.48	1.51	3.05	1.49	1.03	-
Substance use	0.83	1.27	0.27	0.72	2.39	1.79	0.20	0.89	51.59	3 > 1,2,3 1 > 2,4
Behavioral disengagement	1.33	1.52	1.53	1.60	1.56	1.59	1.48	1.49	0.57	-
Self-blame	1.56	1.48	1.52	1.44	1.92	1.84	1.52	1.61	1.04	-
GHQ 28 somatic	8.97	4.84	9.55	4.51	9.85	4.69	8.71	4.91	1.03	-
GHQ 28 anxiety & insomnia	8.9	5.91	9.1	6.05	10.4	6.03	8.6	5.53	1.39	-
GHQ 28 dysfunction	9.0	4.03	9.4	5.08	11.2	5.20	8.8	4.05	4.55	3 > 1,2 *,4
GHQ 28 depression	4.94	4.79	5.96	5.88	7.03	6.03	4.40	4.78	4.21	3 > 1,4
GHQ TOTAL	31.7	16.2	34.0	18.0	38.5	17.7	30.5	16.3	3.43	3 > 1,4

1–2; 1–3; 1–4; 2–3; 2–4—significant differences (*p* ≤ 0.05) between groups, * *p* > 1 statistical tendency
